# Commissioning and quality assurance of the X-ray volume Imaging system of an image-guided radiotherapy capable linear accelerator

**DOI:** 10.4103/0971-6203.41276

**Published:** 2008

**Authors:** K. R. Muralidhar, P. Narayana Murthy, Rajneesh Kumar

**Affiliations:** Department of Physics, Nagarjuna University, Guntur, A.P, India; 1 Indo-American Cancer Institute and Research Center, Hyderabad, Andhra Pradesh, India

**Keywords:** Contrast visibility, image-guided radiation therapy, spatial resolution, X-Ray Volume Imaging system

## Abstract

An Image-Guided Radiotherapy–capable linear accelerator (Elekta Synergy) was installed at our hospital, which is equipped with a kV x-ray volume imaging (XVI) system and electronic portal imaging device (iViewGT). The objective of this presentation is to describe the results of commissioning measurements carried out on the XVI facility to verify the manufacturer's specifications and also to evolve a QA schedule which can be used to test its performance routinely.

The QA program consists of a series of tests (safety features, geometric accuracy, and image quality). These tests were found to be useful to assess the performance of the XVI system and also proved that XVI system is very suitable for image-guided high-precision radiation therapy.

## Introduction

Elekta Synergy (Elekta limited, Crawley, UK) linear accelerator consists of an X-ray Volume Imaging (XVI) system which is designed for kilovoltage (kV) imaging. The XVI system provides image-guided radiotherapy (IGRT) capabilities, which improve the available information to correct for motion and setup errors of the patients. The kV X-ray source and the detector panel are mounted opposite each other across the drum of the digital accelerator. XVI acquires images under the control of the XVI software [Feldkamp-Davis-Kress (FDK) algorithm][1] running on a dedicated XVI workstation.

XVI provides three modes of kV image acquisition: planar view, motion view, and volume view. The cone beam computed tomography (CBCT) provides three-dimensional (3-D) images of patient's soft tissue and bony structure. Image information is very clear in these CBCT images. These images are useful to find the interfractional motion and setup errors by using the 2-D–2-D match and 3-D–3-D match analysis tools. Acquired 2-D and 3-D kV images can be registered with their associated reference images (digitally reconstructed radiographs or planning CT).[2–4] Table corrections are then given by the XVI software in X, Y, Z coordinates. Precise table position can be adjusted by entering the treatment room, when necessary. Elekta Synergy is a high-end equipment and requires not only proper operation but also routine quality assurance (QA) checks. It is essential to carry out routine QA checks in order to keep XVI operating safely, effectively, and reliably. The QA program consists of several tests, covering four components: stability, safety, geometry, and image quality for both 2-D and 3-D images. In this paper, we have described the procedures of the tests included in the QA program and presented the results of the measurements.

## Materials and Methods

The XVI equipment consists of a kV x-ray source (Eureka Rad-92, Varian Sapphire Housing) which generates x-rays that are projected as planar images onto the plate of the kV detector (RID 1640, PerkinElmer Optoelectronics, Wiesbaden, Germany) on the other side of the treatment table. Acquired images are stored on the XVI workstation, which controls the kV generator and the image acquisition process and reconstructs volume images from planar image sets.

When CBCT images are acquired for a patient, the corresponding planning CT and structure sets (anatomic contours) are transferred to the XVI workstation and superimposed on the CBCT images. The patient positioning is analyzed by using 3-D–3-D match. Manual and automated tools are used to align the CBCT images with the planning CT. As with the radiographic mode, the couch shift parameters are downloaded to the linear accelerator, and the couch is moved remotely to correct the patient setup.

### Safety and functionality QA

These tests cover XVI system interlocks, kV imaging arm-touch guard-interlock switch check, and functional movement of the XVI system hardware. The safety QA should be performed on a daily basis before any patient is treated. For proper operation of the imaging system, many of the safety tests with the warm-up procedure for the x-ray tube should be performed.[5,6] Tube warm-up is recommended to prevent premature failure of the x-ray tube. XVI uses preset program for performing tube ‘warm-up,’ to be used each morning, during linear accelerator QA.[7] The most important tests are those for mechanical stability and accuracy of the XVI. These are measured with the help of radiographs taken on daily basis.

System interlocks are checked on room door, kV source arm position, kV imaging panel, beam on, and with termination key. kV imaging arm-touch guard-interlock switch is checked by applying pressure to each of the four corners of the touch guard assembly in turn. Action on each touch guard corner activates interlock, and the corresponding movement stops. Gantry movements are checked when the touch guard is activated. System movement interlock is checked by opening and closing iViewGT and kV imaging panels laterally and longitudinally.

### Geometrical accuracy QA

In geometrical accuracy QA tests, tests for 3-D transverse vertical accuracy, 3-D transverse horizontal accuracy, 3-D sagittal geometric accuracy, 3-D registration accuracy, 2-D geometric accuracy are performed; these are manufacturer-recommended tests with recommended phantoms and test tools.

All CBCT image quality tests use the Catphan 503 phantom, which is provided with the XVI system. Catphan phantom 503 housing is made of solid-cast material made of carbon, oxygen, nitrogen with electron density of 1.04 g/cc. This phantom has four sections with different test modules — CTP401, CTP528, CTP515, and CTP486 — to measure phantom position, alignment, spatial linearity, size of pixel, contrast resolution, and spatial uniformity.

2-D and 3-D tests are used to check geometric accuracy of the data that we get from the XVI. In 2-D–2-D match, length and breadth of the test images can be checked; whereas in 3-D–3-D match, besides the length and breadth, depth can also be checked.

**Figure 1:**
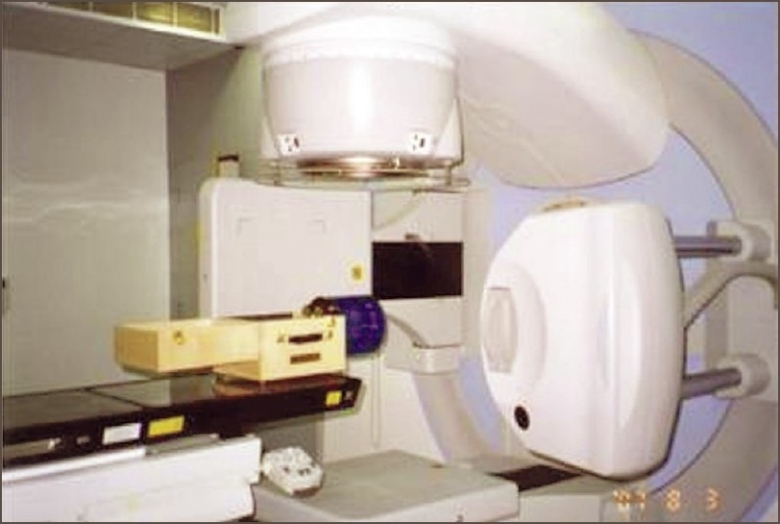
Catphan phantom 503-setup in Linear accelerator

The Catphan phantom is positioned in the linear accelerator [Figure 1] couch by mounting it on the case. Additional weight is required to be placed on the lid to counter-weigh the phantom.[8] The reconstruction matrix was 1024×1024, and the slice thickness was 2.0 mm. From a single scan, all image quality evaluations were made.

**Figure 2:**
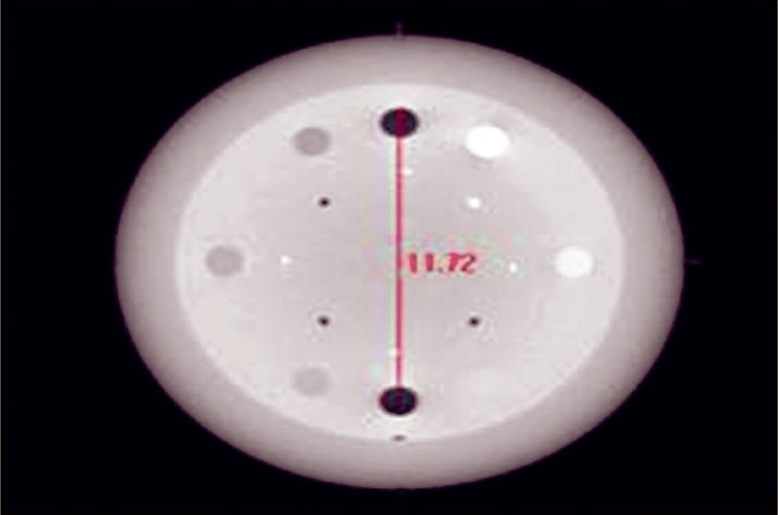
Measuring the distance between the two inserts

In 3-D transverse vertical test, the distance between the centers of the two air inserts [Figure 2] were measured and compared with given data in the contrast resolution module slice. In 3-D transverse horizontal test, the distance between the centers of the Delrdin and the Low Density polyethylene (LDPE) inserts were measured and checked with given data in the contrast resolution module slice. In 3-D sagittal geometric accuracy check, the distance between the first and last ‘dot’ on the Catphan phantom was measured on sagittal view and checked with the given data.

**Figure 3:**
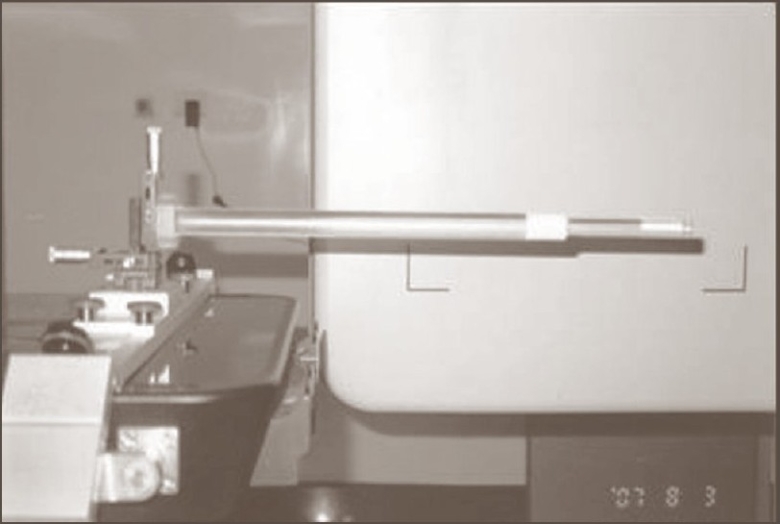
Ball-bearing phantom

**Figure 4:**
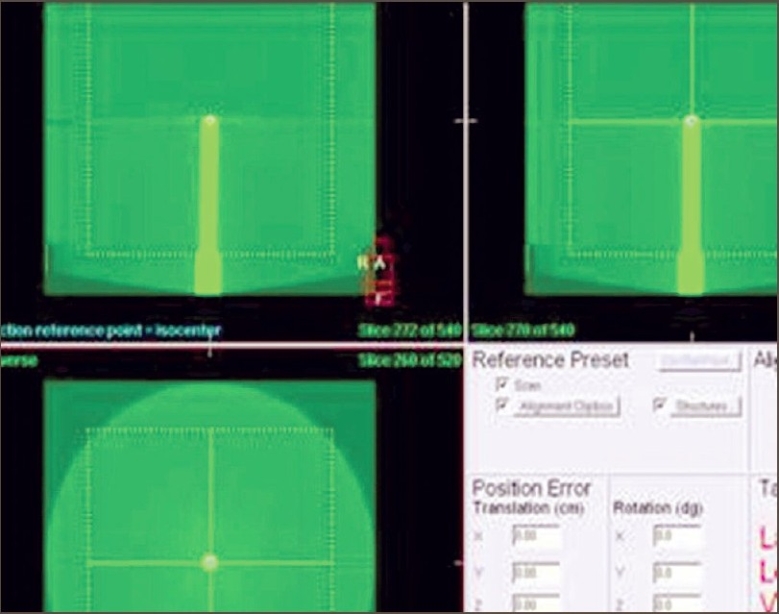
Registraton on transverse, coronal and saggital views

The purpose of the 3-D registration accuracy test is to ensure that the alignment of the isocenters of the kV imaging system and the MV treatment system meets with the specification. For this we used a ball-bearing phantom as shown in Figure 3. This phantom is a long tube made with plastic. An 8-mm diameter steel ball is positioned at the tip of the long tube. Phantom is scanned from −180° to 180°. On transfer of all images to the database, volume view reconstruction is done automatically with Feldkamp-Davis-Kress (FDK) algorithm. Scan was moved manually on registration window so that it matched the plan in all three planes [Figure 4]. After getting the corrections from convert to correction option in the XVI console, table move assistant software highlights the result. Ball-bearing was moved to specified amounts with the help of vernier on the ball-bearing holder. Results are shown in Table 1.

**Table 1:** Results of volume view registration accuracy tests

*Axis*	*Volume view viewer initial correction distance (mm)*
Lateral (Y)	−0.9
Longitudinal (X)	−1.9
Height (Z)	−0.4

In iViewGT, a single exposure was selected and IMRT was delivered. The acquired images were exported from iViewGT to XVI system. Difference between the field center and ball bearing center was recorded. Both the kV and MV registration accuracy tests were carried out. Differences between kV and MV registration values were recorded in Table 2.

**Table 2:** Results of registration accuracy tests

*Axis*	*kV registration accuracy (mm)*	*MV registration accuracy (mm)*	*Difference between KV and MV*
Lateral (Y)	0.2	0.36	0.2
Longitudinal (X)	−0.2	−0.73	0.5
Height (Z)	0.2	−0.35	0.5

The purpose of 2-D geometric accuracy test is to ensure that the display center of the acquired static 2-D kV image matches with the MV radiation isocenter. Single ball bearing was kept at isocenter. Bill ball bearing patent was selected in the control console, which has one treatment field and one kV image. Four planar view images were acquired at gantry angles 0°, 90°, 180°, and 270° with a small field of view. Coordinates (x, y) at the center of the ball bearing and image were recorded. Distance from cross-center to ball bearing center was derived. Distance between cross-center and ball bearing center was measured and recorded in Table 3. It was found that all values were within the specifications (< 4 pixels or < 1.04 mm at isocenter).

**Table 3:** Results of 2-D geometric accuracy test

*Image number*	*Gantry angle (0)*	*Center of ball-bearing*		*Image-center*		*Values within Spec*	

		X	Y	X	Y	X	Y
1	0	505	521	504	518	+1	+3
2	90	507	524	509	520	−2	+4
3	180	501	519	503	515	−2	+4
4	270	509	520	509	516	0	+4

### Imaging tests

In imaging tests, 3-D low-contrast visibility, 3-D spatial resolution, 3-D uniformity, 2-D low-contrast visibility, and 2-D spatial resolution tests were performed, which are manufacturer-recommended tests with their specified phantoms and test tools.

**Figure 5:**
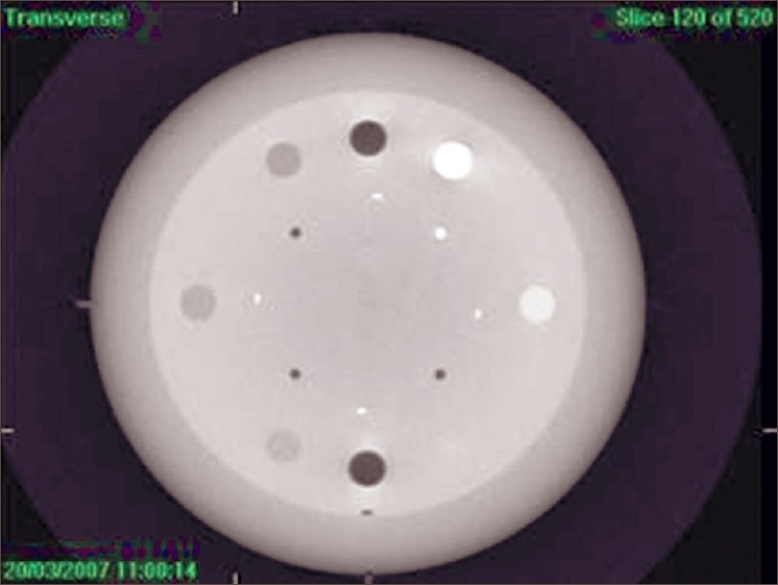
3-D low contrast visibility from Catphan phantom CTP-503

The purpose of 3-D low-contrast visibility test is to check the low-contrast visibility. Catphan phantom CTP-503 was scanned and volume images were acquired. In the contrast resolution module, checking was done to find out where white dots appeared. Pixel values were obtained in transverse window with the help of image probe window. From the image probe window [Figure 5], mean deviation and standard deviation of the contrast polystyrene insert and LDPE insert were obtained. Low-contrast visibility was calculated using the following formula, and the results were tabulated [Table 4].

**Table 4:** Results of 3-D low-contrast visibility test

*Insert*	*Mean pixel value (Mean)*	*Standard deviation (SD)*
Polystyrene	1085.57	17.8
LDPE	827.27	18.65

$$\mathrm{Low}-\mathrm{contrast visibility}=\frac{5.5}{\left[\frac{{\mathrm{Mean}}_{\mathrm{polystyrene}}-{\mathrm{Mean}}_{\mathrm{LDPE}}}{\left[\frac{{\mathrm{SD}}_{\mathrm{polystyrene}}-{\mathrm{SD}}_{\mathrm{LDPE}}}{2}\right]}\right]}$$

**Figure 6:**
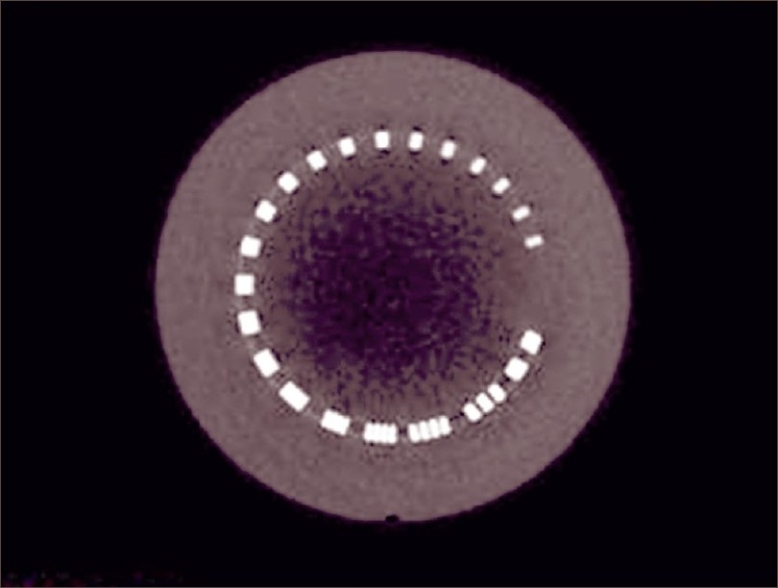
Spatial resolution-transverse

**Figure 7:**
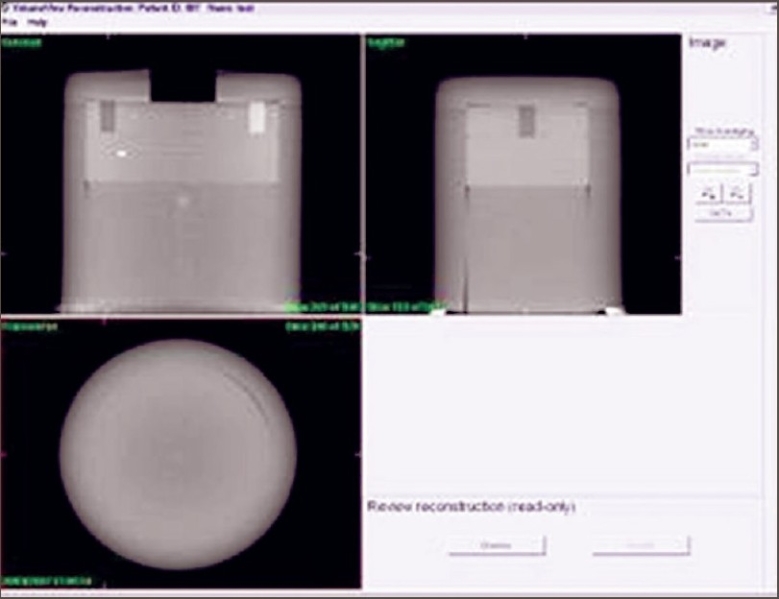
3-D uniformity

In the spatial resolution test, scrolling through the slices was done until the spatial resolution module could be seen, as shown in Figure 6. Image was zoomed and checked. Brightness and contrast were adjusted. Highest numbers of line pairs visible were determined. To find the 3-D uniformity, scrolling through the slices was done until the uniformity module could be seen [Figure 7]. Zoom, contrast, and brightness were adjusted. Mean pixel values were recorded by image probe. Mean pixel values were measured in three other different locations. Maximum differences between any two means were calculated as follows and tabulated [Table 5]..

**Table 5:** Mean pixel values in different locations

*Uniformity section*	*Mean pixel value*
Mean pixel value (center)	646.34
Location 1	646.21
Location 2	645.90
Location 3	646.44

$$\mathrm{Maximum difference between any two means}=\frac{[\mathrm{Mean}(\mathrm{high})-\mathrm{Mean}(\mathrm{low})]*100}{\mathrm{Mean}(\mathrm{high})}$$

We used the Leeds phantom, TOR 18FG (Leeds Test Objects Ltd., North Yorkshire, UK),[9] to monitor both contrast and spatial resolution over time in 2-D imaging systems check. The phantom has 18 discs, each of 8-mm diameter, with contrasts ranging between 16.7% and 0.9%; and 21 bar patterns ranging between 0.50 and 5.00 lp/mm.

The 2-D low-contrast visibility was checked by TOR 18FG Leeds phantom placed on the carbon fiber tabletop at isocenter, with the 1-mm Cu plate positioned on top of the phantom. The ‘top’ arrow was made to point towards the gantry. Low-contrast discs are located peripherally in the phantom, and the spatial resolution module is located in the center.

Planar view images were taken at gantry angle 270°. Brightness and contrast were adjusted such that both brightness and contrast discs were clearly visible. The greater the number of discs that are visible, the better is the low-contrast visibility.

**Figure 8:**
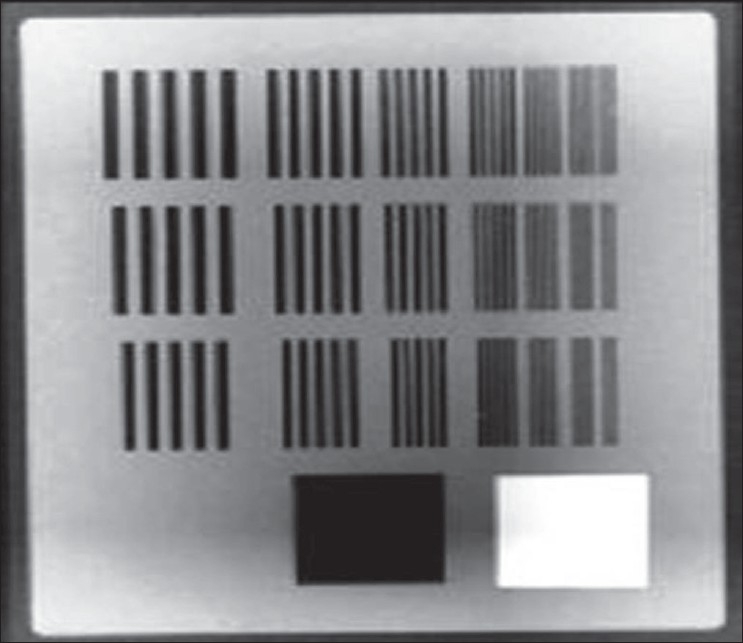
Resolution grid and number of spatial frequency groups

Using the same image acquired previously for contrast, the resolution grid and the number of spatial frequency groups that could be resolved were noted. A frequency group is a group of five independent bars. Numbers of frequency groups visible (Spatial Resolution) were recorded and shown in Figure 8.

## Results

### Safety and functionality QA

Each safety feature (system interlocks check, kV imaging arm-touch guard-interlock switch check, and system movement) was tested daily and we had satisfactory results. They operated and functioned correctly and prevented movement when activated. All the movements of the kV source arm, kV and MV detector panels were satisfactory.

### Geometrical accuracy QA

In 3-D transverse vertical test, the measured distance between the two air inserts is 117.2 mm. The actual distance is 117 mm. The tolerance is <1.04 mm at the isocenter. The difference in measured and actual distance was 0.2 mm, which is well within the specification. In 3-D transverse horizontal test, the measured distance between the Deldrin and LDPE inserts was 116.6 mm. Actual distance is 117 mm. The tolerance is <1.04 mm at isocenter. The difference in measured and actual distance was 0.4 mm, which is well within the specification. In 3-D sagittal geometric accuracy check, the measured distance between the first and last dot was 109.9 mm. Actual distance is 110 mm. The tolerance is <1.04 mm at isocenter. The difference in measured and actual distance was 0.1 mm, which is well within the specification.

In 3-D registration accuracy test, the difference between the kV images to the MV isocenter is within the specification. Image registration specification is less than 1 mm. In 2-D geometric accuracy test, the display center of the acquired static 2-D kV images identifies the MV radiation isocenter. Distance between cross-center and ball-bearing center was measured and recorded in Table 3. All values are found to be within the specifications (<4 pixels at isocenter or <1.04 mm).

### Imaging tests

In the 3-D low-contrast visibility test, the value of low-contrast visibility obtained was 0.38% (which is within the specified value <2%) and it very well meets the required specification. In the 3-D spatial resolution test, the number of line pairs visible was 8 per centimeter, which meets the required specification (>7 line pairs per centimeter). Transverse uniformity meets the required specification (<2%) in 3-D uniformity test. Difference between the two means was 0.02%.

The number of discs visible was 16, which is equal to 1.35% in 2-D low-contrast visibility test. We visually inspected the image and determined the lowest-contrast and lowest-diameter disc that was visible. Manufacturer recommendation is that there should be <3% contrast visibility, or 12 discs should be visible.[10] 2-D spatial resolution meets the required specification (>10 groups, or 1.4 lp/mm). Number of frequency groups visible = 13 (2.2 lp/mm).

## Discussion

The QA programs for XVI are meant to verify whether the safety, mechanical, image, and geometrical parameters are within the prescribed limits or not. Some of the QA programs have to be done on daily basis. Most of the QA programs may be done once in a month or so. The tests use tools provided with XVI. The QA programs for XVI are still being evolved. Here we tried to analyze the stability, safety and functionality, geometrical accuracy, and image tests like contrast and resolution with the help of Catphan phantom 503, ball-bearing phantom, and TOR 18FG Leeds phantom.

We can even apply all QAs related to x-ray machines and CTs; stabilitywise, they are well within limits. Geometric and image tests should also be performed frequently as there is not enough evidence that it would be stable for long durations (8 months). Measurements showed that the mechanical stability of the XVI system is quite high over the 8-month period, when monitored by the radiographic checks of the geometric positioning of the arms.

## Conclusion

The tests were found to be useful in detecting performance of the XVI system that needs recalibration. Use of these tests over an extended period shows that the XVI system has good mechanical reliability and stable image quality. The system is capable of producing images with excellent spatial resolution, as also resolution in high-precision geometry. However, it is important that all tests should be performed on a regular basis within a suggested period to establish guidelines and confidence. These tests also proved that a flat-panel detector is very much suitable for image-guided high-precision radiation therapy.
